# Factors Generating Glucose Degradation Products In Sterile Glucose Solutions For Infusion: Statistical Relevance Determination Of Their Impacts

**DOI:** 10.1038/s41598-017-12296-5

**Published:** 2017-09-20

**Authors:** J. Haybrard, N. Simon, C. Danel, C. Pinçon, C. Barthélémy, F. J. Tessier, B. Décaudin, E. Boulanger, P. Odou

**Affiliations:** 1 CHU Lille, Institut de Pharmacie, F-59000 Lille, France; 2 Univ. Lille, EA 7365 – GRITA – Groupe de Recherche sur les formes Injectables et les Technologies Associées, F-59000 Lille, France; 3 Univ. Lille, EA 2694, 59000 Lille, Cedex France; 4 Univ. Lille, Inserm, CHU Lille, U995 - LIRIC - Lille Inflammation Research International Center, F-59000 Lille, France

## Abstract

Sterilising glucose solutions by heat promotes the generation of a large number of glucose degradation products (GDPs). It has been shown that high levels of GDPs may result in Advanced Glycation End products that have an impact on cellular homeostasis and health in general. If data is available for peritoneal dialysis solutions, little has been published for glucose infusion fluids. It is essential to identify the parameters causing the formation of GDPs and so limit the risk of exposing patients to them. After quantifying both 5-hydroxymethyl-2-furfural, considered as an important indicator of degradation, and 2-furaldehyde, an ultimate GDP of one degradation pathway, in marketed solutions, the aim of this work is to build a model integrating all the parameters involved in the formation rates of these two GDPs: supplier, glucose amount, container material, oxygen permeability coefficient and time-lapse since manufacture. Our results show a good logarithmic relationship between GDP formation rates and time-lapse since manufacture for both GDPs. The amount of GDPs in the glucose solutions for infusion depends on the initial glucose amount, the polymer of the container, the time elapsed since manufacturing and the supplier.

## Introduction

Sterile glucose solutions are commonly used in hospital settings as hydration solutions^1^ or diluents for injectable drugs^2^ as well as for peritoneal dialysis^3^. Considered as inexpensive, safe sources of energy and harmless substances, they are marketed in concentrations ranging from 2.5 to 70 g/100 mL, packed in various containers such as bags, flasks and vials. Primary packaging can be made of materials such as plastic polymers (polyvinyl chloride, polyolefins, multilayers…) or glass. As they are used for intravenous (IV) administration, these solutions are sterile. According to the European Pharmacopoeia^4^, the main sterilisation technique used at the end of their production process is moist heat through a combination of temperature, pressure and time to obtain an adequate microbial lethality rate. F_0_ can be described most simply as the equivalent time required in minutes at 121 °C to produce the same microbiological killing effect as the process used^5^.

**Figure 1:**
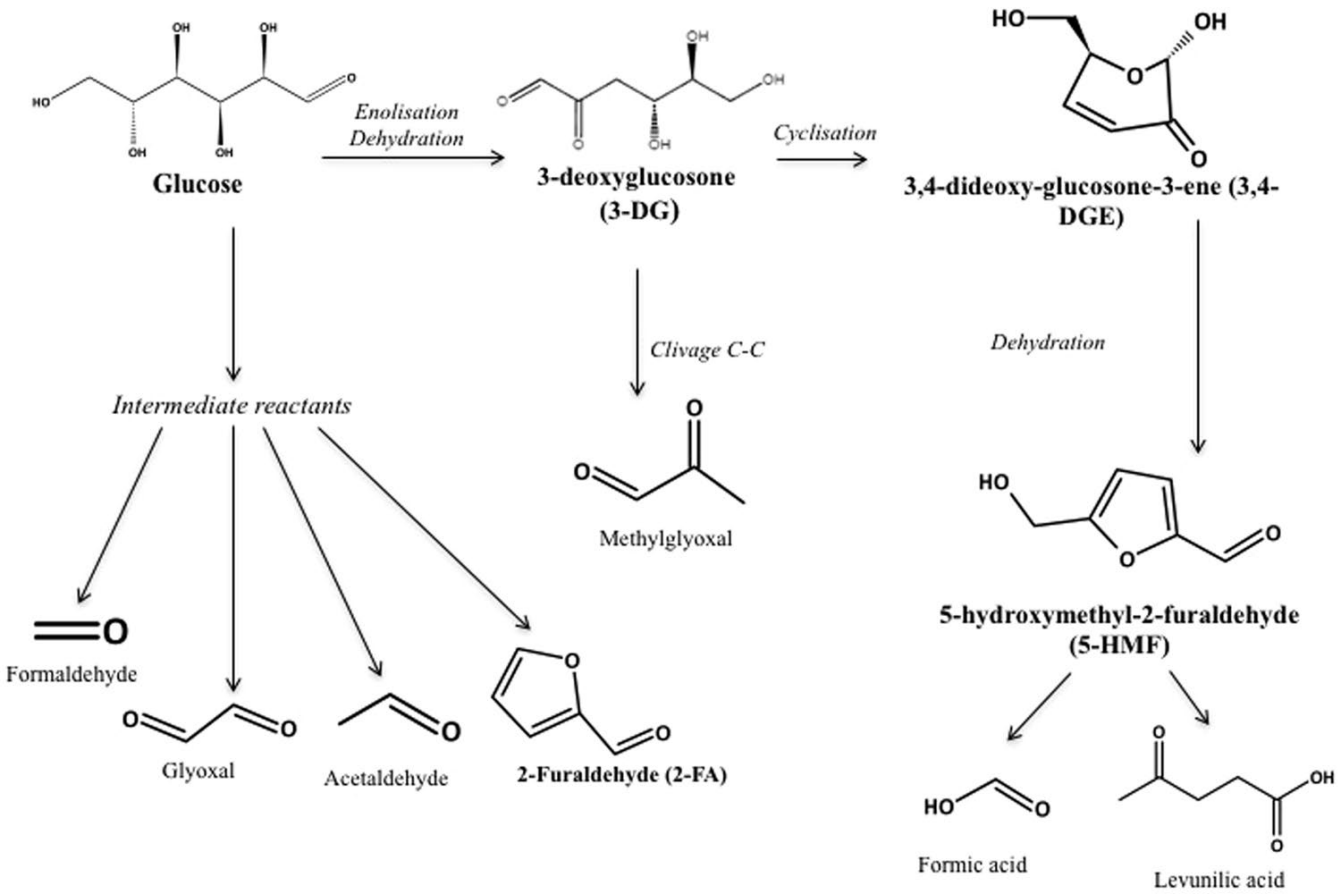
Main glucose degradation pathways. This figure synthetises the main degradation pathways of glucose under high temperature storage conditions^3,10^ or sterilisation^16^.

Heating glucose solutions is known to promote the generation of a large number of glucose degradation products (GDPs)^1,6,7^. Several of these GDPs, such as 3-deoxyglucosone (3-DG), 3,4-dideoxyglucosone-3-ene (3,4-DGE), glyoxal, methylglyoxal, 5-hydroxymethylfurfural (5-HMF), 2-furaldehyde (2-FA), formaldehyde and acetaldehyde, have previously been identified in peritoneal dialysis and infusion fluids^3,8,9^. It has already been demonstrated that GPDs are highly reactive precursors of Advanced Glycation End products (AGEs)^6,10,11^ in proteins. AGEs result from a chemical reaction when reduced carbohydrates (such as glucose) react with amino acids or nucleotides. In 1912, Louis Maillard was the first to describe this non-enzymatic reaction known as the “Maillard reaction”^12^ which is at the origin of a class of products formed spontaneously in nature or in living organisms (e.g. glycosylated haemoglobin)^13^. Among the GDPs cited above, 5-HMF and 2-FA are considered as important indicators of degradation^14,15^ and, according to Linden *et al*.^3^ and Thornalley *et al*.^10^, they may appear in the glucose degradation process under high temperature storage conditions and according to Ulbright *et al*.^16^ under sterilising conditions (Fig. 1).

Although precise thresholds of toxicity are not yet known for GDPs and AGEs administered to chronically-ill patients, it has been shown that high levels of GDPs and AGEs have an impact on cell homeostasis^1,9,17–19^, are involved in oxidative stress^20,21^, are associated with cellular inhibition^22^, induce apoptosis in human leukocytes and renal epithelial cells^1,18,23^, cause degradation of mesothelial cells and peritoneal membrane characteristics^6,23–25^, have an impact on the cardiovascular system^21,26,27^, are associated with an increase in cardiovascular morbidity^28^ and a decline in renal function^29^ or cause kidney damage^1,8,30^. Other studies have shown that the accumulation of AGEs in patients suffering from diabetes mellitus can lead to microvascular complications^6^ such as diabetic retinopathy^20^ or diabetic vascular complications^31^. Very high levels of 5-HMF may lead to acute toxicity^16^.

Little data is at present available on the quantification of GDPs in glucose solutions for intravenous administration^1,14,15,32^. Many questions remain unanswered, particularly regarding the risks involved in the chronic administration of sterile glucose solutions or those associated with chemical interactions with new protein-structured drugs (e.g. antibodies, biosimilars…), which raise the issue of drug glycation before administration^33,34^. Many factors have previously been identified as factors contributing to the formation of GDPs in infusion bags: sterilisation process^5^, storage conditions^7^ or amount of glucose^32^. However, some others have never been tested (e.g. the container) and their impact should be assessed in order to understand how to limit of the formation of GDPs.

Thus, the first objective of this work is to quantify actual amounts of both 5-HMF and 2-FA in marketed glucose IV solutions from different suppliers on the French market and to compare with known toxicological data. The second objective is to build a model integrating all parameters influencing the formation rates of these two GDPs.

## Results

### Measurement of two GDPs in marketed solutions

Both 5-HMF and 2-FA were identified and quantified in each tested solution (Tables 1 and 2). The amounts of 5-HMF vary from 0.20 ± 0.00 mg for the G5% 50 mL of supplier B to 41.21 ± 0.08 mg for G50% 1000 mL of the supplier E. For 2-FA, the variation is observed from 2.42 ± 0.02 µg for G5% 50 mL of supplier E to 654.28 ± 1.04 µg for G15% 1000 mL for supplier D’. An heterogeneity in GDP amounts could be also observed for the same glucose concentration for the same supplier. For example, for the concentration in glucose of 2.5% for supplier A, the amounts range from 0.82 ± 0.00 mg to 3.57 ± 0.03 mg for 5HMF, and from 12.01 ± 0.23 µg to 257.85 ± 3.58 µg for 2-FA. This shows the variability of GDP amounts in our samples.

**Table 1:** Amounts of GDPs in the marketed solutions concerning glucose concentrations of 2.5 and 5%. Each sample was analysed three times. Amounts in glucose degradation products are given as mean ± standard error, expressed in mg for 5-HMF and in µg for 2-FA. G (%): glucose concentration in g/100 ml; Supp.: supplier; Vol.: volume; Cont.: container. Extreme values for one supplier are in italic/bold. *Out of accuracy profile.

G (%)	Supp.	Vol. (mL)	Cont.	5-HMF (mg)	2-FA (µg)	G (%)	Supp.	Vol. (mL)	Cont.	5-HMF (mg)	2-FA (µg)
2.5	A	500	Bag	***0***.***82*** ± ***0***.***00***	***12***.***01*** ± ***0***.13*	5	B	500	Flask	1.92 ± 0.01	48.64 ± 0.27
	A	1000	Bag	***3***.***57*** ± ***0***.***02***	257.85 ± 2.07		B	500	Flask	3.84 ± 0.01	97.76 ± 0.46
	A	1000	Bag	2.81 ± 0.02	***220***.***07*** ± ***0***.***62***		B	1000	Flask	5.60 ± 0.01	150.14 ± 0.12
	B	1000	Flask	1.24 ± 0.01	30.73 ± 0.29*		C	250	Bag	1.33 ± 0.00	25.11 ± 0.34
	E	1000	Bag	2.67 ± 0.00	66.44 ± 1.30		C	250	Bag	***1***.***03*** ± ***0***.***01***	***20***.***69*** ± ***0***.***06***
5	A	50	Bag	***0***.***32*** ± ***0***.***00***	3.40 ± 0.02		C	500	Bag	3.84 ± 0.01	64.98 ± 0.39
	A	50	Bag	0.33 ± 0.00	***3***.***22*** ± ***0***.***01***		C	500	Flask	***4***.***91*** ± ***0***.***01***	***94***.***23*** ± ***2***.***32***
	A	50	Bag	0.35 ± 0.00	3.37 ± 0.01		C	500	Bag	3.57 ± 0.02	68.58 ± 0.38
	A	50	Bag	0.53 ± 0.00	4.46 ± 0.02		C	500	Bag	3.31 ± 0.04	54.35 ± 0.08
	A	100	Bag	0.70 ± 0.01	10.12 ± 0.09		D’	125	Vial	***0***.***48*** ± ***0***.***00***	***11***.***90*** ± ***0***.***02***
	A	100	Bag	0.75 ± 0.01	8.36 ± 0.02		D’	125	Vial	1.01 ± 0.01	20.47 ± 0.17
	A	100	Bag	0.93 ± 0.01	10.53 ± 0.05		D’	250	Vial	2.24 ± 0.01	38.86 ± 0.03
	A	100	Bag	0.98 ± 0.01	10.13 ± 0.01		D’	250	Vial	2.44 ± 0.01	46.09 ± 0.13
	A	250	Bag	0.87 ± 0.00	19.25 ± 0.02		D’	500	Vial	3.54 ± 0.02	62.20 ± 0.02
	A	250	Bag	1.98 ± 0.00	73.36 ± 0.03		D’	500	Vial	3.67 ± 0.01	58.25 ± 0.21
	A	250	Bag	1.08 ± 0.00	25.58 ± 0.02		D’	1000	Vial	5.54 ± 0.01	90.57 ± 0.81
	A	500	Bag	1.87 ± 0.01	33.88 ± 0.14		D’	1000	Vial	***9***.***03*** ± ***0***.***02***	***152***.***87*** ± ***0***.***68***
	A	500	Bag	2.52 ± 0.02	41.55 ± 0.10		E	50	Bag	***0***.***35*** ± ***0***.***00***	***2***.***42*** ± ***0***.02*
	A	500	Bag	***5***.***56*** ± ***0***.***00***	***58***.***66*** ± ***0***.***20***		E	100	Bag	0.65 ± 0.00	7.92 ± 0.03
	B	50	Flask	***0***.***20*** ± ***0***.***00***	***3***.***59*** ± ***0***.***06***		E	100	Bag	1.16 ± 0.00	7.77 ± 0.02
	B	100	Flask	0.24 ± 0.00	4.26 ± 0.15*		E	100	Bag	1.26 ± 0.00	7.97 ± 0.02
	B	100	Flask	***26***.***57*** ± ***0***.***02***	***425***.***43*** ± ***1***.***86***		E	100	Bag	1.30 ± 0.00	7.38 ± 0.08
	B	250	Flask	0.88 ± 0.00	21.39 ± 0.06		E	500	Bag	3.66 ± 0.02	38.90 ± 0.17
	B	250	Flask	0.99 ± 0.00	29.43 ± 0.23		E	500	Bag	***6***.***81*** ± ***0***.***02***	***47***.***20*** ± ***0***.***70***
	B	250	Flask	0.99 ± 0.01	29.27 ± 0.11

**Table 2:** Amounts of GDPs in the marketed solutions, concerning glucose concentrations of 10, 15, 20, 30, 50 and 70%. Each sample was analysed three times. Amounts in glucose degradation products are given as mean ± standard error, expressed in mg for 5-HMF and in µg for 2-FA. G (%): glucose concentration in g/100 ml; Supp.: supplier; Vol.: volume; Cont.: container. Extreme values for one supplier are in italic/bold. *Out of accuracy profile.

G (%)	Supp.	Vol. (mL)	Cont.	5-HMF (mg)	2-FA (µg)	G (%)	Supp.	Vol. (mL)	Cont.	5-HMF (mg)	2-FA (µg)
**10**	A	500	Bag	***3***.***82*** ± ***0***.***06***	104.34 ± 0.05	20	E	500	Bag	21.77 ± 0.02	256.26 ± 1.62
	A	500	Bag	4.09 ± 0.03	***54***.***53*** ± ***0***.***08***	30	B	500	Flask	3.84 ± 0.01	257.92 ± 1.32
	A	1000	Bag	***7***.***57*** ± ***0***.***07***	***122***.***25*** ± ***0***.***12***		D’	500	Vial	***12***.***67*** ± ***0***.***10***	***244***.***63*** ± ***0***.***44***
	B	250	Flask	***2***.***46*** ± ***0***.***01***	***66***.***64*** ± ***0***.***07***		D’	500	Vial	13.81 ± 0.10	263.25 ± 0.76
	B	250	Flask	***2***.***50*** ± ***0***.***01***	***86***.***35*** ± ***1***.***01***		D’	1000	Vial	***34***.***95*** ± ***0***.***22***	***601***.***42*** ± ***1***.***53***
	C	250	Flask	***4***.***61*** ± ***0***.***01***	81.67 ± 0.10		E	250	Bag	***5***.***27*** ± ***0***.***03***	***92***.***14*** ± ***0***.***38***
	C	500	Bag	5.90 ± 0.16	***72***.***75*** ± ***0***.***07***		E	500	Bag	11.44 ± 0.00	160.09 ± 0.05
	C	500	Bag	***6***.***71*** ± ***0***.***02***	***95***.***80*** ± ***0***.***28***		E	500	Bag	13.78 ± 0.06	268.44 ± 1.06
	D	250	Bag	***1***.***62*** ± ***0***.***01***	***28***.***49*** ± ***0***.***08***		E	500	Bag	17.04 ± 0.06	276.70 ± 0.70
	D	250	Bag	1.77 ± 0.01	29.13 ± 0.01		E	500	Bag	***20***.***28*** ± ***0***.***12***	***329***.***51*** ± ***0***.***92***
	D	250	Bag	***2***.***68*** ± ***0***.***03***	***36***.***81*** ± ***0***.***09***	50	B	500	Vial	3.84 ± 0.01	147.80 ± 1.35
	D’	500	Vial	***10***.***06*** ± ***0***.***06***	***300***.***32*** ± ***1***.***01***		E	1000	Bag	41.21 ± 0.08	182.68 ± 3.45
	D’	500	Vial	***12***.***44*** ± ***0***.***14***	***338***.***04*** ± ***0***.***58***	70	B	500	Flask	37.27 ± 0.23	326.09 ± 1.37
	E	100	Bag	***0***.***70*** ± ***0***.***01***	***5***.***50*** ± ***0***.***05***						
	E	100	Bag	1.26 ± 0.02	13.62 ± 0.03						
	E	100	Bag	***1***.***42*** ± ***0***.***00***	***17***.***30*** ± ***0***.***08***						
15	D’	500	Vial	***15***.***95*** ± ***0***.***37***	***337***.***52*** ± ***1***.***10***						
	D’	500	Vial	32.93 ± 0.63	651.83 ± 0.47						
	D’	1000	Vial	***34***.***81*** ± ***0***.***72***	***654***.***28*** ± ***1***.***04***						
	E	500	Bag	***13***.***21*** ± ***0***.***07***	***174***.***82*** ± ***0***.***70***						
	E	500	Bag	13.22 ± 0.21	***145***.***41*** ± ***0***.***58***						
	E	500	Bag	***13***.***71*** ± ***0***.***27***	147.12 ± 19.05						

The mean GDP amounts in all our samples were 6.70 ± 0.58 mg and 114.32 ± 9.00 µg for 5HMF and 2FA, respectively. To compare our data with that in the literature, all amounts were converted into GDP concentrations in each container, giving 13.82 ± 0.79 and 0.23 ± 0.01 µg/mL for 5-HMF and 2-FA, respectively.

### Analysis of the influencing parameters

**Figure 2:**
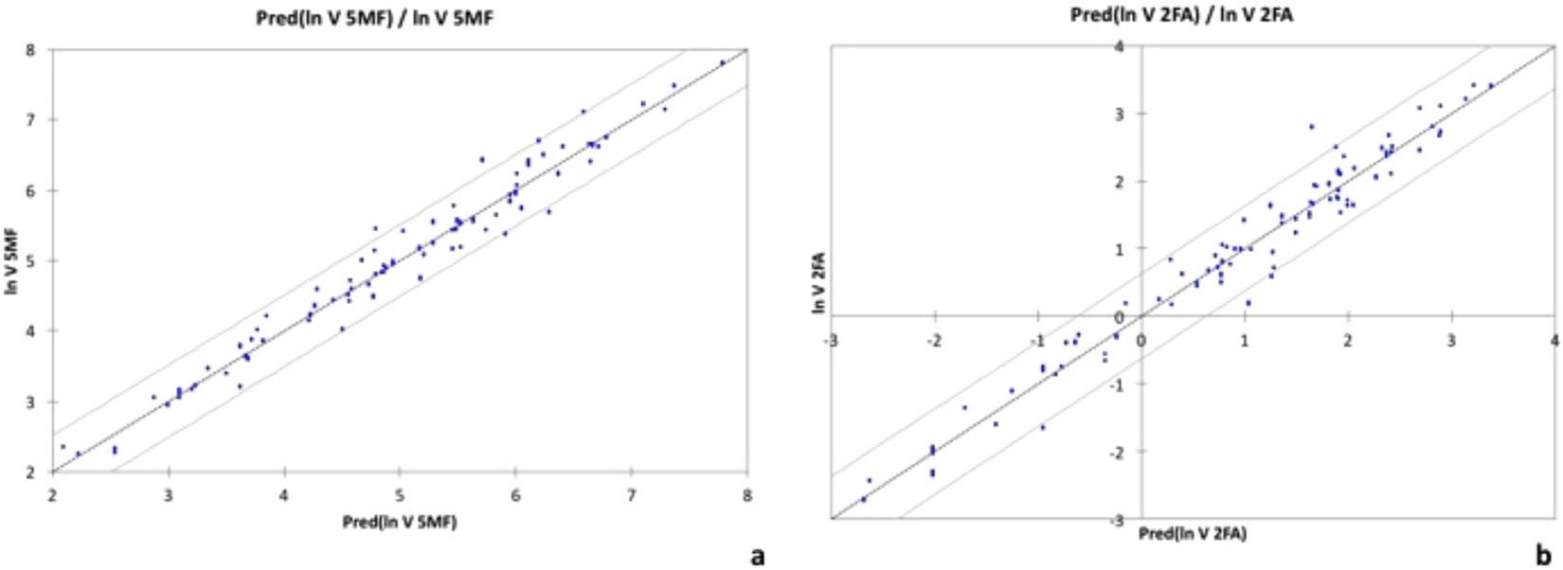
Prediction models for 5-HMF generation (**a**) and 2-FA generation (**b**). Linear relationship between ln(formation rate of GDP) and the prediction of ln(formation rate of GDP). Dots are measured values and continuous grey lines the confidence interval at 95%.

The ANCOVA model showed a good linear relationship between the logarithm of GDP formation rate and time-lapse since manufacture for both GDPs. The simulated and experimental values are very close: r² = 0.966 (p < 0.0001) and r² = 0.962 (p < 0.0001) for 5-HMF and 2-FA, respectively (Fig. 2).

The ANCOVA analysis shows the significant influence of the following parameters on the formation rates of both GDPs: storage time (5HMF: 0.293, p = 0.012 and 2FA: 0.618 p < 0.0001), initial glucose amount in container (5HMF: 2.456, p = 0.005 and 2FA: 4.085, p < 0.001), and permeability to oxygen (1.821 and 1.397, p < 0.001, for 5HMF and 2FA, respectively).

**Figure 3:**
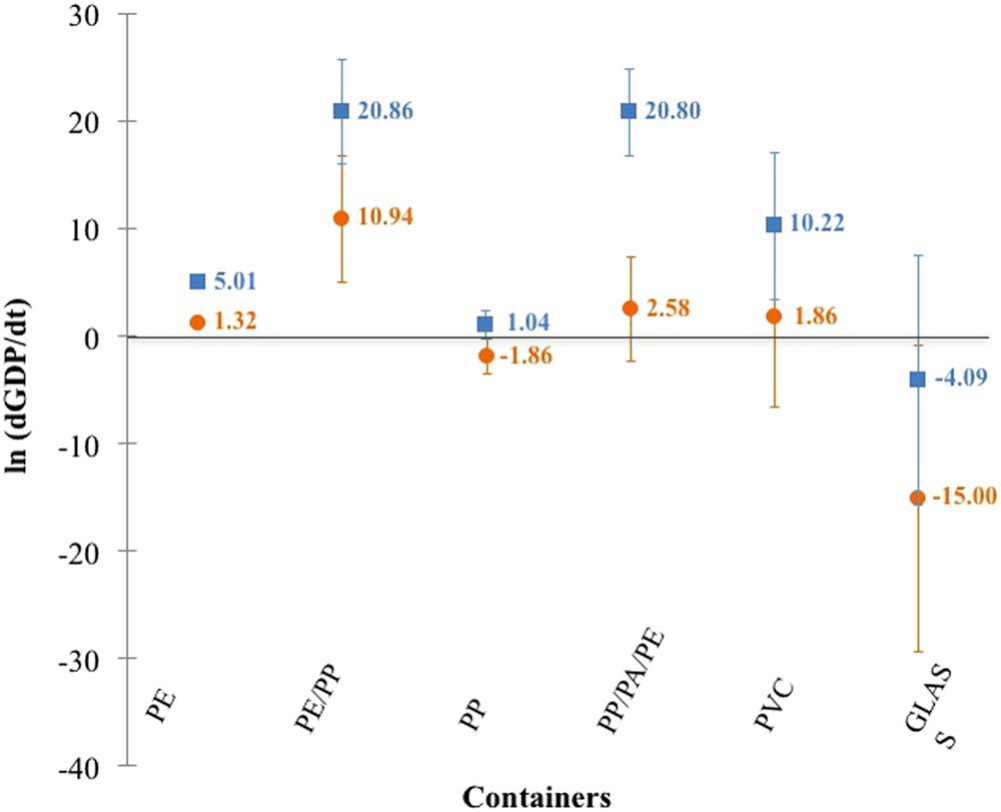
Impact of container material on the formation rate of both 5-HMF (■) and 2-FA (●). Error bars indicate standard deviations.

The influence of container material differs from one material to another. Figure 3 reveals a similar profile on its impact on the formation rate of both 5-HMF and 2-FA. From this data, it is possible to classify container materials according to their ability to limit GDP generation. When considering the influence of container material, GDP formation rate is 1) lower with both PP and glass; 2) not or only slightly influenced with PE, PE/PP and PVC; and 3) higher with multilayer PP/PA/PE.

**Figure 4:**
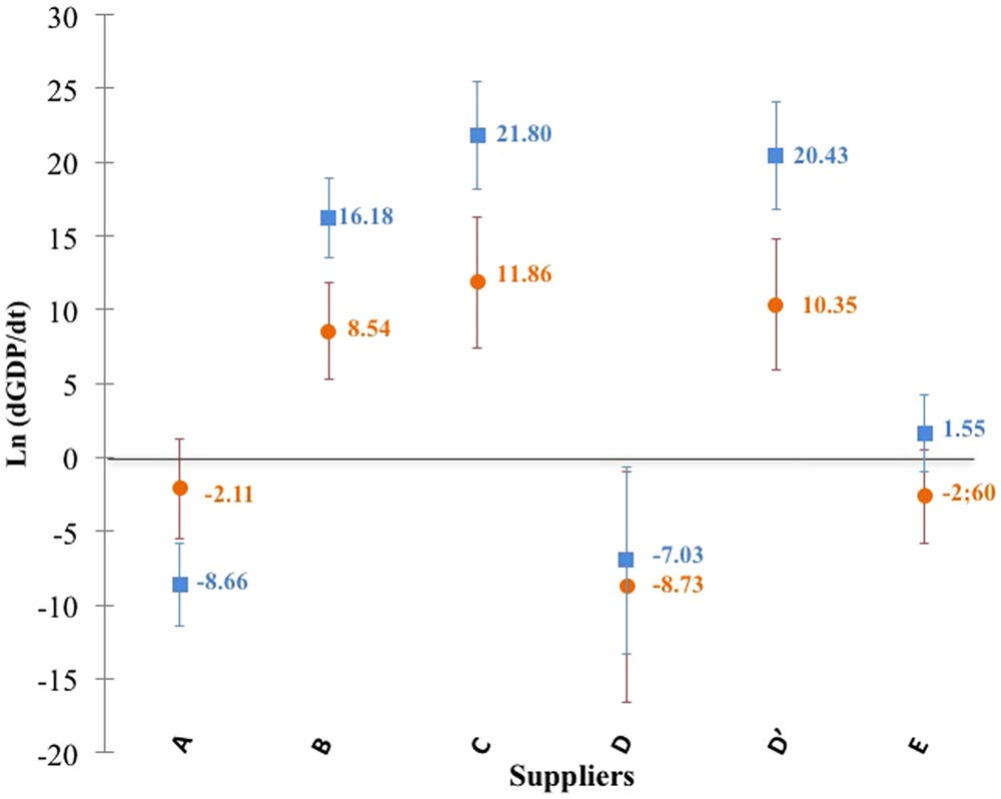
Impact of suppliers on the formation rate of both 5-HMF (■) and 2-FA (●). Error bars indicate standard deviations.

Similarly, the supplier effect was analysed. However, for one supplier (supplier D), two different sterilising conditions were used: one for the plastic container (D) and another for the glass container (D’). For the statistical analysis, we therefore decided to consider this supplier as two different ones (D and D’). So as not to limit the “supplier effect” to only a “container effect”, the ANCOVA analysis also checked the absence of multicollinearity. Results are presented in Fig. 4. Differences in sterilisation techniques have also been highlighted. From this data, suppliers can also be classified according to their ability to limit GDP generation. Suppliers A and D seemed to contribute less to GDP formation (Fig. 4) whereas suppliers C and B seemed to generate more. No significant impact was shown for the other suppliers.

## Discussion

Characterisation of GDPs in injectable glucose solutions has become essential due to increased knowledge about the risks associated with their use. Their presence has already been described, particularly in PD fluids and particularly for 5-HMF. Indeed in this study, both 5-HMF and 2-FA were identified and quantified in all marketed solutions tested. In previously published studies, 5-HMF concentrations ranged from 0.25 to 18.4 mg/L in infusion fluids for glucose concentrations from 2.5 to 30%^1^, or from 7.94 to 36.46 mg/L for glucose concentrations from 10 to 50%^32^. No data is available for 2-FA in infusion fluids, only in peritoneal dialysis fluids. 2-FA concentrations ranged from 38.43 to 134.52 µg/L for glucose concentrations varying from 1.36 to 3.86%^7^ and from 105.69 to 297.86 µg/L for glucose concentrations varying from 1.5 to 7.5%^3^. These values are close to ours: 1.2 and 75.1 mg/L and 23.4 and 779.9 µg/L, for 5-HMF and 2-FA, respectively. The highest values measured in this study for 2-FA, are due to higher glucose concentrations. If only 2.5 and 5% glucose concentrations are taken into account, values are similar to those previously published, with 2-FA concentrations varying from 23.5 to 293.6 µg/L.

Our results demonstrate that both 5-HMF and 2-FA are present in varying quantities in all injectable glucose solutions currently on the French market. Differences from one supplier to another may be explained by different sterilising processes (temperature, pressure and time of sterilization cycle)^1,6,35,36^. All suppliers confirmed the use of the moist heat method according to the European Pharmacopeia, but some did not provide their protocols. After questioning, it appears that they use different temperatures (111 or 121 °C) and F_0_ values (11 to 46). F_0_ is a combination of time and temperature giving equal energy/bacterial lethality^16^. However, the value alone of F_0_ is insufficient; it is important to know the thermodynamic conditions of sterilisation^32^. In their study, Postaire *et al*. demonstrated that the higher the temperature, the higher the GPD formation rate, and independently, the longer the sterilisation time, again the higher the GPD formation rate^32^, which is confirmed in Cook’s study^5^. Kjellstrand *et al*. studied five different F_0_ values and demonstrated that combining a high temperature with a short sterilisation time limited the formation of GDPs^36^. In our study, only one condition was close to those studied by Cook *et al*.^5^: 5% glucose conditioned in 500 mL glass vials, sterilised at 121 °C at an F_0_ value of 20^5^. Applying our equation for a storage time of one month yields an estimated concentration for 5-HMF of 0.662 µg/mL, a value close to previously published results^5^. This confirms the suitability of our model for these conditions.

Apart from sterilisation and storage conditions, we suspected the type of container to be another contributing factor to the formation of GDPs. Indeed, containers with a high coefficient of permeation generate more GDPs than others. This result concords with previously published data showing that high permeation to oxygen can lead to chemical instability of bag contents, especially for easily oxidisable compounds^37,38^. Our results indicate that choosing materials with low permeation to oxygen could be a means of limiting the formation of both 5-HMF and 2-FA.

Finally, this study demonstrates the heterogeneity of patients’ exposure to GDPs according to the glucose solution used. Exposure firstly depends on the solution purchased, which means that it is extremely difficult to determine real exposure to GDPs. To estimate it, we consider that the maximum fluid volume perfused is 2 litres/day^39^. If all infusions are based on glucose, the highest average perfused amount of 5-HMF would be 25.67 ± 1.48 mg/day. Matzi *et al*. showed that an oral daily intake of 720 mg of 5HMF over 10 days had no apparent toxicological effect^40^. The absolute bioavailability of 5-HMF is estimated at 72%^41^, so 720 mg/day oral intake is equivalent to an approximate IV dose of 520 mg/day. This data confirms that patients hospitalised for a short period and receiving dextrose infusions at low volumes are at a low risk of toxicity, as suggested by a previously published study^16^. Little data is currently available on toxicity associated with the chronic infusion of these products, especially in particularly fragile populations (e.g. neonates, patients suffering from critical conditions or patients receiving long-term IV therapy with glucose solutions). It has previously been shown that GDPs and AGEs may disrupt cellular homeostasis^1,8,9,11,17,42^ or lead to clinical disturbances^1,8,21,26,27,30^, and so further *ex vivo*, *in vivo* and clinical studies are required to establish the toxicity profile of such solutions.

The originality of this work is to indicate that, by using a statistical model, factors other than sterilisation, storage conditions and initial glucose amount may influence the formation of GDPs during storage:

1. GDP formation rate is well correlated with storage duration (between 4 and 61 months).
2. Oxygen permeability has a significant influence on the formation rate of both GDPs. PP and glass limit the formation of GDPs (Fig. 4). The multilayers PP/PA/PE, PE/PP and PVC are unable to limit or prevent the generation of GDPs. The impact of PE is not significantly different from 0.

To conclude, this work shows that the following factors influence GDP formation: initial amount of glucose, supplier, mean oxygen permeability coefficient, type of container materials and storage duration since manufacture. Until toxicity studies have been carried out on patients, some measures could be applied to reduce their risk of exposure: the use of glucose solutions with low concentrations conditioned in glass vials and stored for only a short period of time.

## Methods

### Chemicals and reagents

Methanol (HPLC – grade), 100% glacial acetic acid, anhydrous potassium dihydrogenophosphate, 5-HMF and 2-FA were purchased from VWR International S.A.S (Fontenay-sous-Bois, France). Pure water was produced with an ultrapure water system (Ultrapure water, Purelab UHQ, ELGA). Anhydrous glucose was obtained from Inresa (Bartenheim, France).

### Analytical method

The analyses were carried out by an HPLC system (Shimadzu, Noisiel, France) equipped with an autosampler (SIL-20AC XR) with a 100 µL injection loop, a diode array detector (SPD-M20A) and a system of double flow-rate pump solvent module (LC-20AD XR). A column oven (CTO-20AC) was used to maintain the column (Alltech Apollo^TM^ C_18_ column, 5 µm, 150 × 4.6 mm I.D. (Fisher Scientific, Illkirch, France) at 25 °C. All data was analysed with LabSolution software (Shimadzu, France).

**Figure 5:**
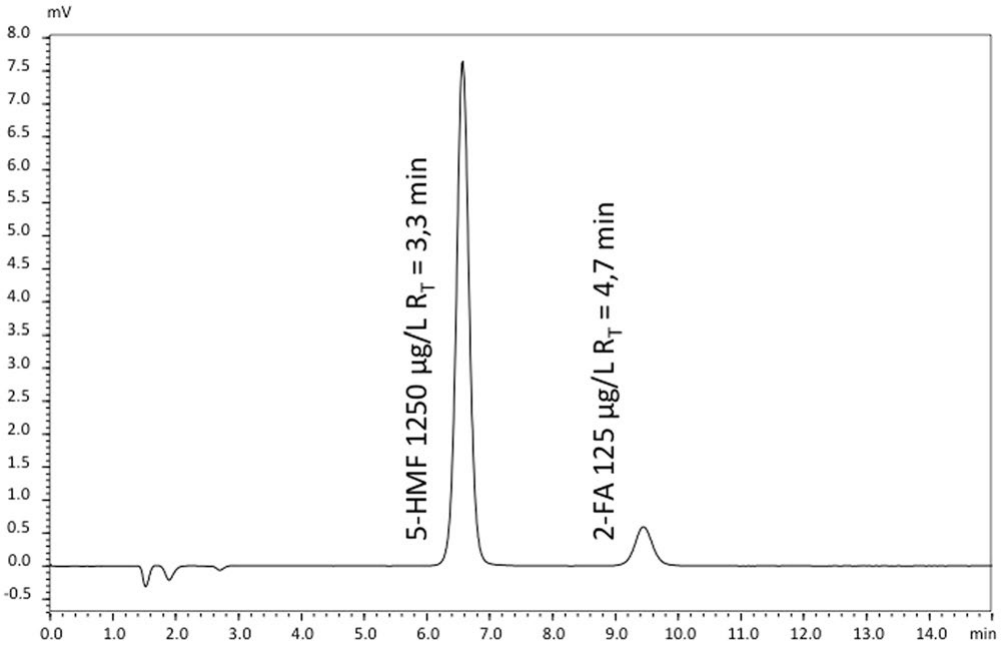
Chromatogram of a solution containing 5-HMF and 2-FA. The concentrations of 5-HMF and 2-FA are 1250 µg/l and 125 µg/l, respectively (Rs = 6.5).

The mobile phase consisted of a mixture (10%/90% - v/v) of methanol and phosphate solution (1.5 g, 0.011 mmol for 1 litre) adjusted to pH 2.95 with acetic acid. Flow-rate was set at 2 mL/min. Detection wavelength for both GDPs was fixed at 284 nm following literature guidelines^11^. A chromatogram is depicted in Fig. 5.

Concentrations were chosen for calibration points based on previously published data^1^ and results of pre-validation steps in our laboratory. Calibration ranges were 500–2000 µg/L and 50–200 µg/L for 5-HMF and 2-FA, respectively. The method was validated by an analysis of variance (ANOVA). Range for validation assay, regression parameters, limits of detection (LOD) and of quantification (LOQ) were 36 and 12 µg/L, and 72 and 24 µg/L for 5-HMF and 2-FA, respectively. Validation data are summarized in Table 3.

**Table 3:** Regression parameters, LOD and LOQ for each GDP.

GDP	Range (µg/L)	r^2^	Slope	y-Intercept	LOD (µg/L)	LOQ (µg/L)
5-HMF	500–2000	0.999	92686.87	−1349.29	36	72
2-FA	50–200	0.988	12.95	7.83	12	24

A volume of 100 μL glucose solution was injected into the HPLC-UV system without pre-treatment with a 100-μL injection loop. If after dosing, the back-calculated concentration was outside the validated ranges, the solution was diluted with pure water to reach a concentration within the validated ranges and re-analysed. High glucose concentrations (≥ 30 g/100 ml) were systematically diluted with pure water to avoid injecting too viscous a solution. Each glucose solution was measured in triplicate.

Each concentration measured in each container was transformed into amounts by multiplying the concentration by the container’s nominal volume. The nominal volume was used because it is the only volume communicated by all the suppliers. Finally, the GDP rate was determined by dividing the amount by storage duration. For the analysis of glucose infusion fluids, all results were anonymised and are given as mean ± standard error.

### Analysis of marketed solutions

The analysis involved 84 glucose solutions coming from 5 suppliers (Baxter, Lavoisier, MacoPharma, BBraun and Fresenius) at 8 concentrations (2.5, 5, 10, 15, 20, 30, 50 and 70 g/100 mL), 6 volumes (50, 100, 125, 250, 500 and 1000 mL) and in 3 types of containers: bags, flasks and vials. Six different materials: plastic polymers (polyvinyl chloride (PVC), polyethylene (PE), polypropylene (PP), multilayer PP-PE and multilayer PP-polyamide (PA)-PE and glass were used as raw materials for the containers. All solutions were stored from 4 to 61 months at room temperature, in industrial cardboard boxes, in the warehouse of hospital pharmacy, located in the basement of the building. The detail of each solution tested is presented in Table 4.

**Table 4:** Description of tested solutions. All suppliers confirmed the use of the moist heat method but some did not provide their protocol: A: Not communicated, B: Not communicated, C: F_0_ ≥ 11, D: F_0_ ≥ 15 (estimated between at 42 and 45), D’: F_0_ ≥ 15 (estimated at 20), E: F_0_ = 15.

Supplier	N	Conc (%)	Volume (ml)	Type of Container	Type of container material	Time-lapse since manufacture (months)
A	20	2.5–5–10	50–100–250–500–1000	Bag	PE/PA/PP	7–9–10–12–14–15–16–17–20–31–32–33–34–51
B	15	2.5–5–10–30–50–70	50–100–250–500–1000	Flask – Vial	PE - Glass	4–5–6–7–8–11–17–18–25–29–36
C	9	5–10	250–500	Flask – Bag	PE – PP	15–16–18–20–34–43–44
D	3	10	250	Bag	PP	13–19
D’	16	5–10–15–30	125–250–500–1000	Vial	Glass	10–13–15–18–20–23–25–28–31–33–46–50–54–58–61
E	21	2.5–5–10–15–20–30–50	50–100–250–500–1000	Bag	PE/PP - PVC	11–15–16–17–18–20–23–26–29–31–37–41–56

### Statistical model

Two factors have been added to those already described in the literature.

1. Suppliers (S): A to E (i.e. sterilisation process^5^);
2. *Initial glucose amounts (G*_0_): 2.5, 5, 6.5, 10, 12.5, 25, 50, 75, 100, 150, 250, 300, 350 and 500g^32^;
3. *Time-lapse since manufacture (t)*. This parameter was defined as the delay between measurement and manufacturing dates, estimated by subtracting the expiry date from official shelf life^43^. The shelf lives used in this study were validated by each pharmaceutical supplier for each batch.
  Since permeability to oxygen was suspected to be a prominent factor in the formation of GDPs, we added the following factors to the model:
4. *Materials (M)*: PVC, PE, PP, multilayers (PE-PP and PE-PA-PP) and glass;
5. Mean *oxygen permeability coefficient (P)*. This parameter was estimated from previously published data^44^. The mean oxygen permeability coefficient for plastic flasks made of PE was estimated at 25 × 10^−10^ (cm^3^.mm)/(cm^2^.s.cm of mercury)^44^. For bags made of either PA, PVC or PP, it was estimated at 0.2 × 10^−10^, 0.6 × 10^−10^ and 8 × 10^−10^ (cm^3^.mm)/(cm^2^.s.cm of mercury), respectively^44^. For glass vials, it was fixed at 0 because glass is gasproof. These oxygen permeability coefficients have been referred to the surface of containers and to atmospheric pressure.

To determine a potential relationship between influencing parameters and GDP formation rate, an analysis of covariance (ANCOVA) model was used. However, the relationship between the two variables must be linear to use the ANCOVA model and this was obtained by logarithmic transformation. The complete model used in this study can be summarised in the following equation:

1 $$\begin{array}{c}\mathrm{ln}({\rm{dGDP}}/{\rm{dt}})={\rm{C}}+\backslash \mathrm{alpha}\backslash \mathrm{times}\,\mathrm{ln}({\rm{G}}\_0)+\backslash \mathrm{beta}\backslash \mathrm{times}\,\mathrm{ln}({\rm{P}})+\backslash {\rm{gamma}}\backslash {\rm{times}}\,{\rm{M}}+\\ \quad \quad \quad \quad \quad \quad \quad \backslash \mathrm{delta}\backslash \mathrm{times}\,{\rm{S}}+\backslash {\rm{varepsilon}}\backslash {\rm{times}}\,{\rm{I}}-{\rm{k}}\backslash {\rm{times}}\,{\rm{t}}\end{array}$$

where G_0_, P, M, S, t are the previously cited parameters and I, the interaction between the different coefficients. α is the coefficient for each amount of glucose; β, the coefficient for each case of oxygen permeability; γ, the coefficient for each material; δ, the coefficient for each supplier and ε the coefficient for interaction.

A two-tailed type I error < 0.05 was considered for statistical significance. Analyses were conducted using XLSTAT^®^ software (v2014.5.02, Addinsoft, Paris, France).
